# Kinetics of the Neutralizing Antibody Response to Respiratory Syncytial Virus Infections in a Birth Cohort

**DOI:** 10.1002/jmv.23696

**Published:** 2013-08-27

**Authors:** CJ Sande, MN Mutunga, EA Okiro, GF Medley, PA Cane, DJ Nokes

**Affiliations:** 1 Kenya Medical Research Institute (KEMRI), Centre for Geographic Medicine Research (Coast)Kilifi, Kenya; 2 School of Life Sciences and WIDER, University of WarwickCoventry, United Kingdom; 3 Public Health EnglandLondon, United Kingdom

**Keywords:** RSV, neutralizing antibody dynamics, immunity

## Abstract

The kinetics of respiratory syncytial virus (RSV) neutralizing antibodies following birth, primary and secondary infections are poorly defined. The aims of the study were to measure and compare neutralizing antibody responses at different time points in a birth cohort followed-up over three RSV epidemics. Rural Kenyan children, recruited at birth between 2002 and 2003, were monitored for RSV infection over three epidemic seasons. Cord and 3-monthly sera, and acute and convalescent sera following RSV infection, were assayed in 28 children by plaque reduction neutralization test (PRNT). Relative to the neutralizing antibody titers of pre-exposure control sera (1.8 log_10_ PRNT), antibody titers following primary infection were (i) no different in sera collected between 0 and 0.4 months post-infection (1.9 log_10_ PRNT_,_ *P* = 0.146), (ii) higher in sera collected between 0.5 and 0.9 (2.8 log_10_ PRNT, *P* < 0.0001), 1.0–1.9 (2.5 log_10_ PRNT, *P* < 0.0001), and 2.0–2.9 (2.3 log_10_ PRNT, *P* < 0.001) months post-infection, and (iii) no different in sera collected at between 3.0 and 3.9 months post-infection (2.0 log_10_ PRNT, *P* = 0.052). The early serum neutralizing response to secondary infection (3.02 log_10_ PRNT) was significantly greater than the early primary response (1.9 log_10_ PRNT, *P* < 0.0001). Variation in population-level virus transmission corresponded with changes in the mean cohort-level neutralizing titers. It is concluded that following primary RSV infection the neutralizing antibody response declines to pre-infection levels rapidly (∼3 months) which may facilitate repeat infection. The kinetics of the aggregate levels of acquired antibody reflect seasonal RSV occurrence, age, and infection history.

## INTRODUCTION

A recent review highlighted the significant burden of severe acute lower respiratory tract disease attributable to respiratory syncytial virus (RSV) [Nair et al., 2010]. Understanding the duration of neutralizing antibody responses following natural exposure will inform future control strategies by providing estimates of the duration of a key correlate of protective immunity.

Acquired and maternally derived neutralizing antibodies to RSV appear to correlate well with protection from severe disease [Eick et al., 2008; Glezen et al., 1981; Piedra et al., 2003] and re-infection [Hall et al., 1991; Lee et al., 2004]. Despite development of these responses, RSV does infect individuals repeatedly [Hall et al., 1976; Henderson et al., 1979] suggesting that protective immunity is of short duration or that the virus through antigenic variation is capable of escaping protective immune responses or both mechanisms are at play. The duration of protection from infection and disease provided by both maternally-derived and acquired neutralizing antibody in early infancy is not well quantified, although a recent study shows that the risk of re-infection is significantly reduced for 6 months following primary infection [Ohuma et al., 2012]. Although previous studies have shown that serum antibody responses acquired following primary infection with RSV decline to pre-infection levels within a year [Ochola et al., 2009; Welliver et al., 1980] antibody detection in these studies has been based on enzyme linked immunosorbent assays (ELISAs) or indirect immunofluorescent antibody techniques. These methods detect the total antibody response and not the neutralizing response which may be a better correlate of protective immunity.

The effect of variation in population-level virus transmission on population-level immunity has not been investigated exhaustively. Studies on the temporal relationship between neutralizing antibodies of maternal origin and population-level virus transmission have shown that population-level neutralizing antibodies decline in the absence of exposure and increase following an increase in virus transmission at the population-level [Stensballe et al., 2009], implying that a decline in herd immunity may establish the conditions necessary for the spread of the virus in the population.

In the current study, the duration of neutralizing antibody responses to RSV was investigated and the relationship between population-level transmission and the kinetics of the neutralizing antibody response were analyzed. Determination of the duration of the neutralizing antibody response following natural exposure will provide important information on the potential effectiveness of future vaccine programs in reducing virus transmission and consequently the burden of RSV disease.

## MATERIALS AND METHODS

Nasal samples from which the test viruses were derived were inoculated onto HEp-2 cells, incubated at 33°C and examined daily for development of cytopathic effect. An immunofluorescent antibody test (IFAT; Millipore, Billerica, MA) was used to verify isolation of the virus in culture. Virus quantitation was done using the plaque assay while neutralizing antibody titers were determined using the plaque reduction neutralization assay. Detailed descriptions of these assays have been published elsewhere [Sande et al., 2013]. Neutralizing antibody titers were determined as neutralizing dose 50 (ND_50_) values using the Spearman-Karber method [Cohen et al., 2007] and the results expressed as plaque reduction neutralization titers (PRNTs). A log_10_ transformation was used to normalize the data for statistical analyses. Representative local RSV A (Kil/A/2006) and B (Kil/B/2008) viruses isolated in 2006 and 2008, respectively were used as the test viruses. Neutralizing antibodies to both strains were measured and a mean titer calculated. This mean value was used in analysis.

The study used archived serum and nasal wash samples collected from a birth cohort of children recruited between 2002 and 2003 in the rural District of Kilifi on the Kenyan coast [Nokes et al., 2004,^2008^]. Recruitment was undertaken in two phases: the first phase between January and May 2002 and the second phase between December 2002 and July 2003. In total 635 infants were recruited in the birth cohort study [Nokes et al., 2008]. At the time of delivery, a cord blood sample was taken, followed by blood samples scheduled at 3-monthly intervals until each child had experienced three RSV epidemics or was lost to follow-up. During home or clinic surveillance, nasal washes were collected from children who displayed symptoms of acute respiratory infection and detection of RSV done using IFAT. An acute blood sample was collected as soon as possible after diagnosis of RSV infection and a convalescent blood sample was collected about 1 month later. Further study design details have been published elsewhere [Nokes et al., 2008]. The present study included 28 children from the birth cohort from whom at least eight serum samples had been collected over the course of follow-up. All had a virus confirmed primary infection, while nine had virus confirmed secondary infection. Serum neutralizing antibodies were measured in the acute and convalescent sera of the children who were followed-up as well as in the cord blood sample and in the routine 3 monthly sera. A negative (pre-exposure) control group was used for the purpose of comparison consisting of sera collected up to 6 months before a primary infection from children who were older than 5 months of age at the time of collection. All participants in this study provided written informed consent prior to sample collection. Ethical approval for this study was provided by the Kenya Medical Research Institute Ethical Review Committee.

The dynamics of neutralizing antibodies at the cohort-level were analyzed by calculating the mean titers in successive time intervals (strata) each of three calendar months duration. Stratification was carried out independently for the two birth cohort phases. The relationship between cohort-level antibody dynamics and population transmission of RSV was assessed by overlaying the RSV incidence data onto the mean cohort-level neutralizing antibody titer data. A correlate of the temporal incidence of RSV in the community was obtained from continuous surveillance of RSV admissions to Kilifi District Hospital with RSV-associated pneumonia [Nokes et al., 2009]. The development of the neutralizing response with age was assessed by comparing the observed mean cohort-level peak titers at different time points over the duration of follow-up. The time strata with the highest mean titer following the start of an epidemic was considered to have the peak neutralizing antibody titer for that epidemic.

Data were analyzed using Stata (version 11, StataCorp; College Station, TX). For the purpose of calculating the duration of the neutralizing response, the start of the host response was assumed to coincide with the date of collection of an RSV positive nasal sample. It was assumed further that antibody responses had declined to pre-infection levels if there mean levels were not statistically different from the mean pre-exposure control titer.

The duration of the neutralizing antibody response following primary infection was determined using a regression model with clustered sandwich estimation to account for repeated measurements. In this model the neutralizing antibody titers were the dependent variable while the number of months before or after infection and age were the explanatory variables. Differences in mean cohort-level neutralizing titers at different time points were analyzed using a regression model in which neutralizing titers were the dependent variable and the different time strata were the explanatory variables.

## RESULTS

**Figure 1:**
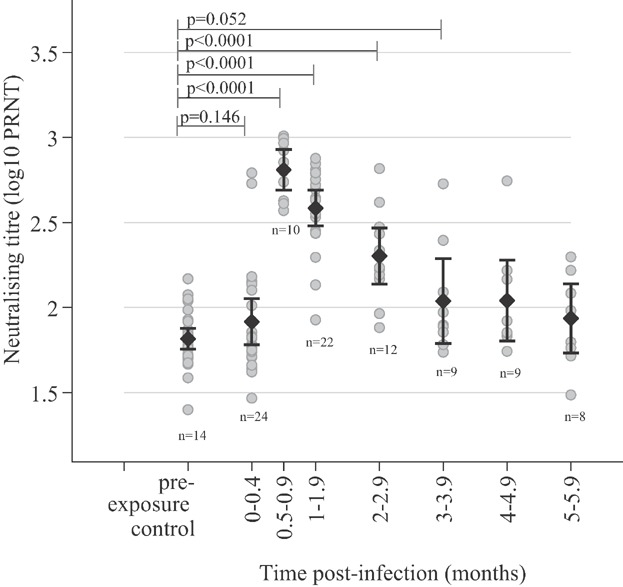
The dynamics of the neutralizing antibody response following primary infection were determined by comparing the mean pre-exposure control titer to titers in sera collected at 0–0.4, 0.5–0.9, 1–1.9, 2–2.9, 3–3.9, 4–4.9, and 5–5.9 months after infection. The gray circles indicate the distribution of neutralizing antibodies; the diamond markers indicate the mean titer in each group while the whiskers denote 95% confidence intervals about the mean. The *P*-values indicate whether the difference between the mean pre-exposure control and mean titers at different time points post-infection is statistically significant. The number of samples at each time point is shown below the respective distributions.

The time course of the primary neutralizing antibody response was estimated by comparing antibody titers at different time points post-infection to the neutralizing titers in the pre-exposure control (Fig. 1). There was no difference between the mean pre-exposure control titer (1.8 log_10_ PRNT) and the mean titer in sera collected between 0 and 0.4 months after infection (1.9 log_10_ PRNT, *P* = 0.146). The mean titer increased significantly at 0.5–0.9 months post-infection (2.8 log_10_ PRNT, *P* < 0.0001), 1.0–1.9 months post-infection (2.5 log_10_ PRNT, *P* < 0.0001) and at 2.0–2.9 months post-infection (2.3 log_10_ PRNT, *P* < 0.0001). There was no difference between the mean pre-exposure control titer and the mean titer at 3.0–3.9 months post-infection (1.8 log_10_ PRNT vs. 2.0 log_10_ PRNT, *P* = 0.052).

**Figure 2:**
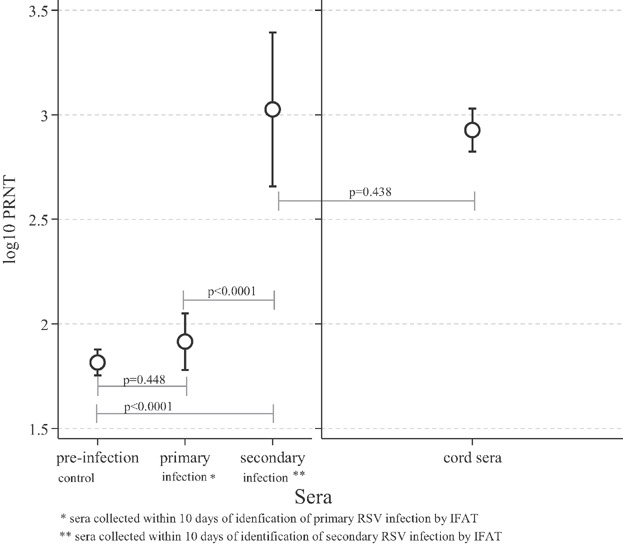
The mean neutralizing antibody titer (open circles with corresponding 95% confidence intervals) in the pre-exposure control is compared to the mean titer in sera collected within 10 days of identification of both primary and secondary infecting virus. Mean titers at the primary and secondary infection stage are also compared. Comparison is also made between the mean acute titers at the secondary infection stage and cord titers. The lines connecting the different groups being compared indicate whether differences in mean titer are statistically significant.

The kinetics of the early response to primary and to secondary infection was evaluated by comparing the neutralizing responses in sera collected within 10 days of the identification of the infecting viruses to the mean pre-exposure control titre. There was no difference between the mean pre-exposure control titer and the mean titer in sera collected within 10 days of the identification of primary infection (1.8 log_10_ PRNT vs. 1.9 log_10_ PRNT, *P* = 0.448). On the other hand, the mean titer in the sera collected within 10 days of the identification of secondary infection (3.02 log_10_ PRNT) was significantly greater than the mean pre-exposure control titer (*P* < 0.0001) as well as the mean titer in the sera collected within 10 days of the identification of primary infection (*P* < 0.0001). No difference was found between the early secondary response and the mean neutralizing antibody level in cord sera (*P* = 0.438). These data are shown in Figure 2.

**Figure 3:**
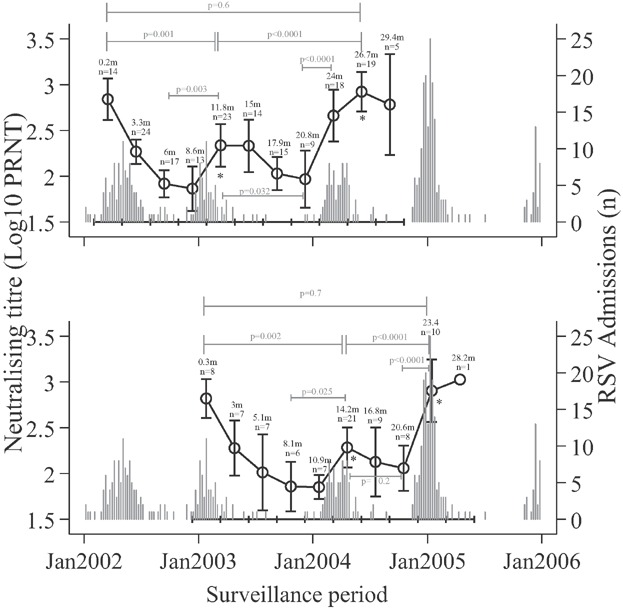
The relationship between cohort-level neutralizing antibody dynamics and population-level virus transmission was determined by overlaying mean neutralizing titers in successive three calendar month strata within phase 1 (top panel) and phase 2 (bottom panel) of the birth cohort onto RSV weekly case data identified through pediatric pneumonia surveillance at Kilifi district hospital. The open circles and corresponding whiskers indicate the mean titer within a particular stratum and 95% confidence intervals. The numbers above each stratum indicate the mean age and the total number of samples tested in that stratum. The vertical bars indicate weekly admission totals of infants admitted with RSV related pneumonia over the surveillance period (right axis). The graduated line at the bottom of the RSV incidence bars indicates stratum boundaries. The *P*-values above the gray lines connecting different strata indicate whether there is a statistically significant difference between mean titers of the groups being compared. Asterix symbols (*) indicate the strata that were considered to have the highest mean titers (peak titers) following an epidemic.

The first 6–8 months of life were characterized by a decline in maternally derived neutralizing antibodies against a background of increased population-level virus transmission (Fig. 3). Increased virus transmission in the second epidemic coincided with significant increases in the cohort-level titers of both phase 1 (*P* = 0.003) and 2 (*P* = 0.025) as shown in Figure 3 and correspondingly, the decline in population-level virus transmission was associated with a significant decline in cohort-level titers in phase 1 (*P* = 0.03) but not phase 2 (*P* = 0.2). Increased virus transmission in the third epidemic was also associated with significant increases in cohort-level titers in cohort phases 1and 2 (*P* < 0.0001).

The development of the neutralizing response with age was examined by comparing the mean cohort-level peak titers in different time strata. The peak titers in the second epidemic experienced by phase 1 and 2 respectively were significantly lower than the peak (presumably maternally derived) titers in the first epidemic (*P* = 0.001 and *P* = 0.002 respectively) and lower than titers in the third epidemic (*P* < 0.0001 and *P* < 0.0001 respectively). There was no difference between the peak titers in epidemics 1 and 3 of phase 1 (*P* = 0.6) and 2 (*P* = 0.7) respectively. These data are shown in Figure 3.

## DISCUSSION

In this study the kinetics of the RSV neutralizing antibody response in a birth cohort followed-up over the course of 3 RSV epidemics with both active and passive case detection were examined. The results show that natural primary infection in infants induced a strong neutralizing antibody response that declined to pre-infection levels at between 3 and 4 months post-infection as shown in Figure 1. The rate of development of this primary response was lower compared to the corresponding secondary response since the mean neutralizing titer in sera collected within 10 days of the identification of the secondary infecting virus, was significantly greater compared to the corresponding primary response as well as the pre-exposure control. In contrast there was no difference between the mean neutralizing titer in cord sera and the mean titer collected in sera collected within 10 days of the identification of secondary infection. The rapid decline of the primary neutralizing antibody response suggests that the ability of the virus to cause secondary infection may be related to the short duration of primary neutralizing antibody immunity.

This reduction in protection from the serum neutralizing antibody response demonstrates that it is not antigenic variation per se that permits multiple re-infection with RSV. It has been suggested previously that re-infection may be due to viral antigenic differences [Pothier et al., 1987] and recent work has shown that in most cases the virus strains causing primary and secondary infections in an individual are genetically distinct [Agoti et al., 2012]. On the other hand, previous work has shown that neutralizing antibody immunity induced by natural infection is group but not genotype specific [Sande et al., 2013], which suggests that antigenic heterogeneity, as determined using serum cross neutralization assays, is only functional at the group but not at the genotype level. The current results suggest that the primary neutralizing antibody response is short-lived and may partially contribute to the susceptibility to re-infection by RSV in young children.

The overlay of RSV incidence data onto the neutralizing antibody data within successive time strata in the birth cohort showed that while the first epidemic to be experienced by either phase did not induce a significant cohort-level response (presumably due to inhibitory or obscuring effects of maternal antibodies [Murphy et al., 1986]), the second and third epidemics were both accompanied by significant rises in mean cohort-level titers. On the other hand, a decline in virus transmission led to a corresponding decline in cohort-level neutralizing antibodies. This study did not examine the kinetics of the neutralizing antibody response in older children and adults. It is possible that natural infection in older individuals may induce an antibody response whose longevity varies from that of the relatively young population represented in this study. Future studies should consider investigating the dynamics of neutralizing responses not only in infants and young children but also in other age groups in order to provide additional information on the relationship between herd antibody dynamics and recurrence of RSV in the population.

In this study, the effect of age and repeated exposure on the magnitude of the response generated following natural infection was examined by comparing the peak cohort-level titers in each of the three epidemics during which infants were followed-up. Peak titers in the first epidemic were assumed to be of maternal origin since samples in the first epidemic were obtained from very young infants in whom maternal antibodies are typically present at relatively high titers. On the other hand, as shown in Figure 3, the peak titers in the second and third epidemics were most likely to be reflective of acquired responses since they were preceded by relatively low titers and occurred at an age when maternal antibodies are generally considered to have waned to minimal levels. When the three peaks were compared, it was found that the peak titer in the first epidemic was significantly greater than the peak titer in the second epidemic, but was not significantly different from the third epidemic peak, while the peak titer in the second epidemic was significantly lower than the third epidemic peak. In addition, as illustrated in Figure 2, the rate of development of secondary antibody response was significantly greater than the primary response rate. These results strongly suggest that after the decline of maternal antibodies, the infant neutralizing response progressively increases in magnitude with successive exposure. This is supported by studies among Native American children in the United States, which have shown evidence of a higher risk of admission to hospital following infection in comparison to the general population [Bockova et al., 2002]. In these studies it was reported that the risk of hospital admission is related inversely to the titer of neutralizing antibodies [Eick et al., 2008], suggesting that the increase in magnitude of the neutralizing antibody response with age is linked to the reduced risk of severe disease with age. Taken together, these data suggest that repeated exposure primes the development of high titers of neutralizing antibodies, potentially reducing the likelihood of development of severe disease. This is supported by previous work that showed that infants with RSV re-infections shed virus for a significantly shorter duration in comparison to infants experiencing their first infection [Okiro et al., 2010].

In summary, the current work describes the kinetics of the neutralizing antibody response in infants followed-up in a birth cohort. The data presented show that RSV neutralizing antibodies in infants decline rapidly following natural infection. The data presented in this paper suggest that if future vaccines induce neutralizing antibody immunity whose longevity is of comparable duration to that induced by natural infection, there will be need for the administration of booster doses of the vaccine in order to maintain neutralizing antibodies at protective levels.
